# Lentinan enhances the antitumor effects of Delta-like 1 via neutrophils

**DOI:** 10.1186/s12885-022-10011-w

**Published:** 2022-08-25

**Authors:** Haiyan Xu, Ziwei Qi, Qi Zhao, Jiao Xue, Jiaxing Zhu, Yan He, Guirong Liu, Songbing Qin

**Affiliations:** 1 Department of Radiotherapy, the First Affiliated Hospital of Soochow University, Suzhou, 215006 Jiangsu China; 2 Department of Medical Oncology, the Second People’s Hospital of Lianyungang, Lianyungang, 222000 Jiangsu China; 3 Hematology Center, Cyrus Tang Medical Institute, the Collaborative Innovation Center of Hematology, State Key Laboratory of Radiation Medicine and Protection, Soochow University, Suzhou, 215123 Jiangsu China

**Keywords:** Lentinan, Delta-like 1, Neutrophils, Breast tumor, Lung cancer

## Abstract

**Background:**

Selective activation of Delta-like 1 (DLL1)-Notch signaling is a new approach to activate CD8^+^ T cell and suppress tumor growth, while the efficacy remains modest. Lentinan (LNT) is a clinically used immunomodulation agent. Thus, we hypothesized that LNT could improve the efficacy of DLL1.

**Methods:**

The effects of LNT combined with DLL1 on tumor growth were evaluated by growth curve and tumor weight in EO771 breast and LAP0297 lung tumor models. The impacts on immune cells and gene expression in tumor tissues were determined by flow cytometry, qPCR. Neutrophil depletion was used to investigate the mechanism of the combination therapy on tumor growth. The data sets were compared using unpaired student’s *t*-test or ordinary one-way ANOVA.

**Results:**

LNT treatments additively improved the antitumor effects of DLL1 in EO771 breast tumor growth. Remarkably, LNT treatments synergistically enhanced the suppression of DLL1 on LAP0297 lung tumor growth, resulting in tumor regression. Mechanically, the combination of LNT and DLL1 interventions not only promoted the accumulation and activation of CD8^+^ T cells, but also increased intratumoral CD45^+^CD11b^+^Ly6G^+^ neutrophils. Reduced neutrophils by anti-Gr1 antibody administrations reversed the improved antitumor effects by LNT treatments in LAP0297 lung tumor. These results suggest that LNT treatments improve the inhibition of DLL1 on tumor growth via neutrophils.

**Conclusions:**

Our findings indicates that LNT and DLL1 may induce synergistical antitumor immunity via simultaneous modulating lymphoid and myeloid cell populations regardless of the type of tumor, providing a potential new strategy to potentiate cancer immunotherapy.

**Supplementary Information:**

The online version contains supplementary material available at 10.1186/s12885-022-10011-w.

## Background

Tumor is an organ-like tissue that results from the co-evolution of malignant cells and their immediate environment. Understanding the crosstalk between cancer cells and their environment is critical to developing more effective therapies. The dialogue between cancer cells and their environment involves syntactic and paracrine signaling through many different pathways, for example EGFR signaling and Notch signaling. In murine, Notch signaling pathway has four Notch receptors (Notch1, Notch2, Notch3, and Notch4) and four ligands (Jagged1, Jagged2, Delta-like 1 (DLL1), and DLL4) [1]. Notch signaling pathway plays critical and diverse roles in cancer, both tumor promotion and tumor suppression [2, 3]. Therefore, targeting on the Notch signaling in cancer has been extensively studied over the past 20 years, but the biological effects of Notch signaling on tumor initiation and progression remain not fully understood. Some reported the tumorigenic activity of this pathway [4], while others reported the tumor suppressive function of this pathway [5]. Activation of the Notch pathway can trigger carcinogenic and tumor suppressive functions, which seem to depend on specific cellular and tissue environments.

Notch signaling also plays crucial roles in regulating the differentiation and activation of immune cells. Selective activation of DLL1/Notch signaling by multivalent clustered DLL1 induced anti-tumor immune response and suppressed lung cancer growth [6]. Overexpressing DLL1 in bone marrow progenitor cells can also activated CD8^+^ T cells and inhibit fibrosarcoma tumor growth [7]. Moreover, elevated DLL1 in the tumor microenvironment (TME) upregulated transcription of granzyme B and interferon γ (IFN-γ) and slowed down the growth of breast and lung tumors [8, 9].

Although selectively stimulating DLL1/Notch signaling has been shown to suppress tumor growth, only a small part of tumors regressed and most tumors kept growing, just at a relatively slower growth rate. This is similar with immune checkpoint therapy, which can induce durable antitumor immunity and prolong survival, but only in a small fraction of cancer patients [10, 11].

Combinational therapy is considered as a feasible strategy to improve cancer immunotherapy [12]. Increased DLL1 levels in the TME sensitized anti-cytotoxic T lymphocyte-associated protein 4 (anti-CTLA4) treatment in its resistant tumors, leading to tumor regression and longer survival [8]. Besides lymphocytes, innate immune cells also involve in shaping tumor immunity. Many solid tumors, such as breast and lung tumor, are infiltrated with neutrophils. Tumor-infiltrated neutrophils can exert antitumoral or pro-tumoral effects [13, 14]. Recent studies suggested that neutrophils can be trained by polysaccharide, such as β-glucan or nanomaterials, to induce antitumor immunity and potentiate immune checkpoint therapy [15, 16]. Lentinan (LNT) is another kind of polysaccharide extracted from *Lentinus edodes*, and has been used as an immunopotentiator in the clinic [17, 18]. As the activation of DLL1/Notch signaling stimulates adaptive immune cells, simultaneously stimulating innate immune cells might enhance its antitumor efficacy. Therefore, we investigated the effects of LNT on DLL1-mediated tumor growth inhibition in both orthotopic breast tumor and ectopic lung tumor models. Our data showed that LNT treatments can synergize with DLL1 overexpression to induce LAP0297 lung tumor regression via neutrophils.

## Methods

### Reagents

The Lentinan (LNT, 1 mg, 2005801A), a gift from Nanjing EASEHEAL Pharmaceutical Co., Ltd. (Nanjing, China), is provided as powder in a penicillin bottle. LNT was dissolved in saline (0.9% NaCl) right before in vivo administration.

### Cell lines

The EO771 is a breast adenocarcinoma cell line originally isolated from a spontaneous tumor in C57BL/6 mouse and was purchased from CH3 Biosystems (New York, USA). The LAP0297 is a lung alveolar–bronchiolar carcinoma isolated from a spontaneous tumor in FVB mouse and was generated by Dr. Peigen Huang at Massachusetts General Hospital (Boston, USA) [19]. DLL1 overexpressing cell lines (EO771-L1 and LAP0297-L1/LAP-L1) or cell lines with control vector (EO771-R1 and LAP0297-R1/LAP-R1) were generated by Dr. Yuhui Huang at Soochow University [8]. Tumor cells were cultured in Dulbecco’s modified Eagle’s medium (DMEM) (GIBCO) supplemented with 10% heat-inactivated fetal bovine serum (FBS) (GIBCO) and 1% penicillin and streptomycin (GIBCO) at 37 °C in a humid incubator containing 5% CO_2_. Cell cultures were frequently monitored for mycoplasma contamination, and only mycoplasma-negative cells were used for experiments.

### Tumor models

Female C57BL/6 mice (6–8 weeks old) and female FVB mice (6–8 weeks old) were purchased from the Shanghai Laboratory Animal Center (Shanghai, China). All of the mice were housed in the specific pathogen-free (SPF) condition. C57BL/6 mice were orthotopically inoculated with 2 × 10^5^ cells of EO771-R1 or EO771-L1 into the third mammary fat pad [8]. FVB mice were subcutaneously inoculated with 2 × 10^5^ cells of LAP0297-R1/LAP-R1 or LAP0297-L1/LAP-L1 on the right flanks [8]. When tumors reached 4–6 mm in diameter, mice bearing tumors were randomly divided into appropriate groups and subjected to Lentinan or saline (0.9% NaCl) treatments [17]. Tumor volume (mm^3^) was estimated by using the following formula: tumor volume = (long axis) × (short axis)^2^ × π/6. All animal studies were approved by the Institutional Laboratory Animal Care and Use Committee of Soochow University. All experimental methods were conducted in accordance with the Animal Care and Use Regulations of China.

### Flow cytometry analysis

Mice-bearing tumors were intracardially perfused with PBS. Tumor tissues were isolated and single cell suspensions were prepared by using the digesting DMEM medium containing collagenase type 1A (200 U/ml), hyaluronidase (1000 U/ml) and DNase (20 U/ml). Rat anti-mouse CD16/CD32 monoclonal antibody was added into the single-cell suspensions before other antibody staining. After staining, cells were washed with cold flow buffer (1% BSA, 0.1% NaN_3_ in PBS), and 7AAD reagent (eBioscience) was added (5 μl/tube) prior to running the flow analysis. Flow cytometry data were acquired on a Gallios flow cytometer (Beckman) and analyzed with a Kaluza software (version 1.3). The following fluorescence-labeled or isotype-matched anti-mouse antibodies were used: CD8-FITC, CD4-PE, CD69-PE-Cy7, CD44-APC, CD62L-APC-Cy7, CD4-Alexa Flour 700, Gr1-APC-Cy7, CD25-APC-Cy7, Ly6G-FITC, F4/80-PE, CD45-BV421, and CD11b-BV510 (BioLegend) [8, 17].

### In vivo anti-Gr1 antibody administration

When LAP0297-R1 or LAP0297-L1 lung tumors reached 5–6 mm in diameter, mice bearing tumors were randomly divided into four groups, which were treated with an isotype-matched control saline (0.9% NaCl), 1 mg/kg LNT, 200 μg anti-Gr1 antibody (clone RB6-8C5, Bio-X Cell), or LNT plus an anti-Gr1 antibody [15, 17]. Gr1 is the myeloid differentiation antigen, also known as Ly-6G/Ly-6C. Saline, anti-Gr1 antibody or LNT plus an anti-Gr1 antibody were administered into mice via *i.p.* injection every 2 days. The treatment duration was 7–14 days.

### Quantitative real-time PCR

Total RNA from tumor tissues was isolated by a MicroElute Total RNA kit (Omega), followed by cDNA synthesis with a RevertAid First Strand cDNA Synthesis Kit (Thermo Scientific). The levels of related mRNA were determined by using a high-throughput fluorescence quantitative PCR meter (LightCycler480 II) (Roche). The primers (Table 1) were specifically designed to avoid nonspecific amplification by one half hybridizing to the 3′end of one exon and the other half hybridizing to the 5′end of the adjacent exon. β-actin was used as a reference gene to calculate the differences in gene expression (fold change) [17].

**Table 1:**
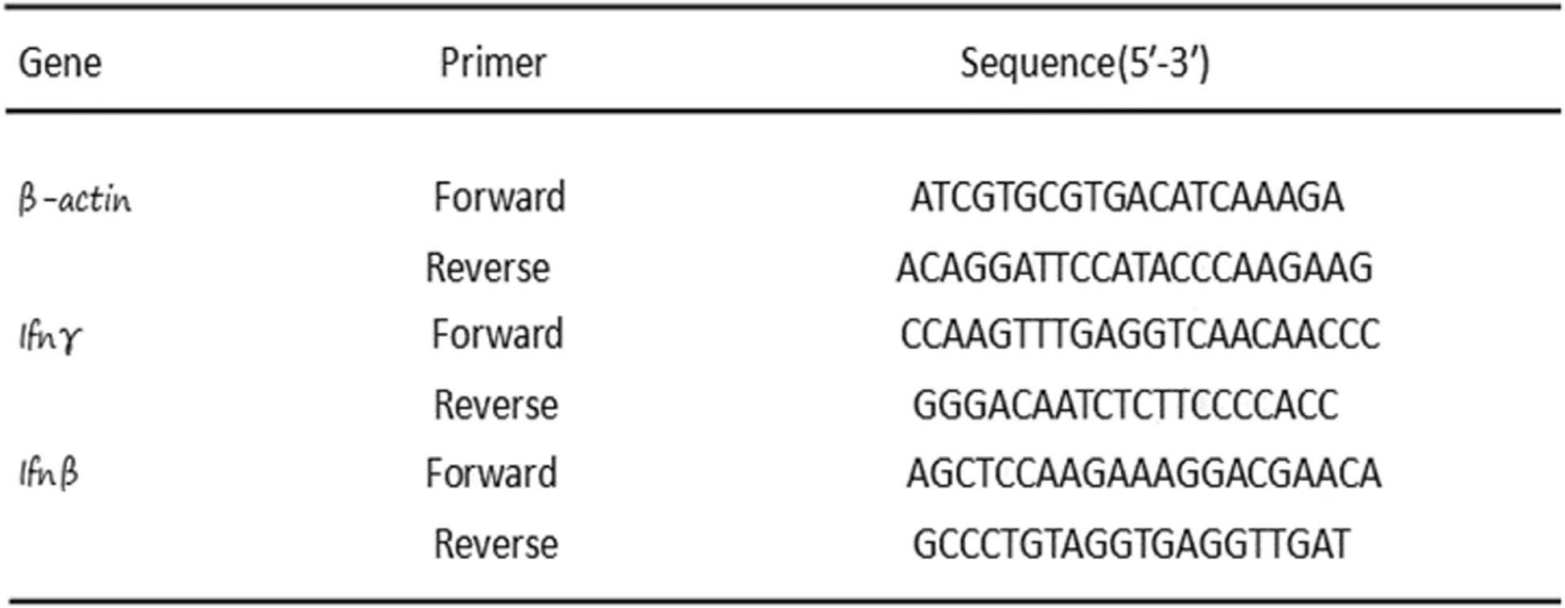
Primers used for qPCR analysis

### Statistical analysis

Statistical analyses were performed using a Prism statistical software (version 7, GraphPad Software, Inc.). Unpaired two-tailed student’s *t*-tests were used to determine the statistical differences between two groups. Ordinary one-way ANOVA was used to assess the differences when more than two groups were compared. Data were presented as mean ± standard error of the mean (SEM). The results were considered as statistically significant at *P* < 0.05 (*). *P* values lower than 0.01 or 0.001 were indicated as “**” or “***”, respectively.

## Results

### Relatively low dose LNT treatments can inhibit EO771 breast tumor growth

Tumor immune evasion is a hallmark of cancer [7, 20, 21]. LNT and DLL1 have been shown to suppress the growth of several types of cancer and the effects have been largely attributed to immune stimulation [20, 22–24]. Whether LNT combined with DLL1 intervention can enhance the inhibitory effect on the growth of cancers. To determine the impact of LNT combined with DLL1 on tumor growth, we firstly evaluated the dose effects of LNT treatments on EO771 breast tumor. We treated tumor-bearing mice with 0.5, 1.0, 2.0, or 4.0 mg/kg/daily LNT for 9 days (Fig. S1a). LNT treatment at the dose of 0.5 mg/kg/daily inhibited EO771 breast tumor growth, compared with control group (Fig. S1b). In order to optimize the usage of LNT, we reduced the dosage frequency and treated tumor-bearing mice with 0.5, 1.0, 2.0 mg/kg LNT every other day for 9 days (Fig. S2a). Our data showed that LNT treatments at the dose of 1 mg/kg exhibited better antitumor effects, compared to control group (Fig. S2b). Together, these data show that only a relatively low dose LNT treatments exhibit tumor growth inhibition. Based on these results, we chose 1.0 mg/kg every other day as the standard LNT treatment regimen for EO771 breast tumors in the rest of this study.

### LNT enhances the antitumor effects of DLL1 in EO771 breast tumor

**Fig. 1:**
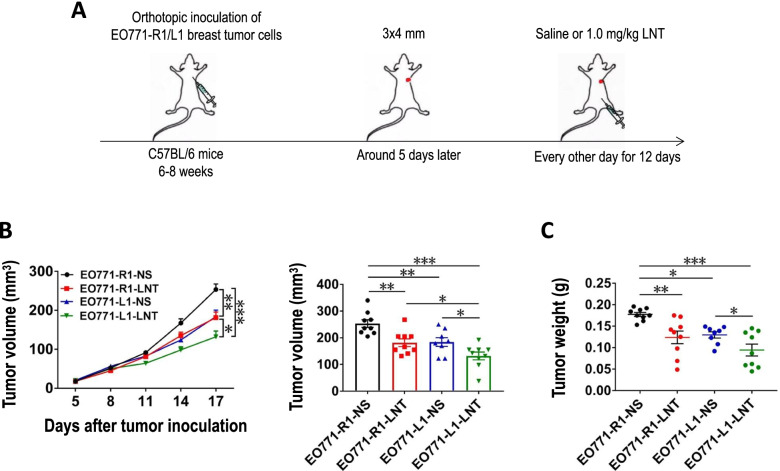
LNT enhanced the antitumor effects of DLL1 in EO771 breast tumors. **a** Experimental design: C57BL/6 mice were orthotopically inoculated with 2 × 10^5^ EO771-R1 or EO771-L1 breast cancer cells on the mammary fat pad. When tumors reached 3 × 4 mm in diameter, mice were randomly divided into 4 groups and received *i.p.* injection of saline or 1.0 mg/kg LNT every other day for 12 days; **b **Tumor growth curves were recorded and tumor volume was calculated at the end of the treatments; **c** Tumor weight was measured at the end of the treatments. NS: control group treated with saline. All data in this report are presented as means ± SEM. * *p* < 0.05, ** *p* < 0.01, *** *p* < 0.001

**Fig. 2:**
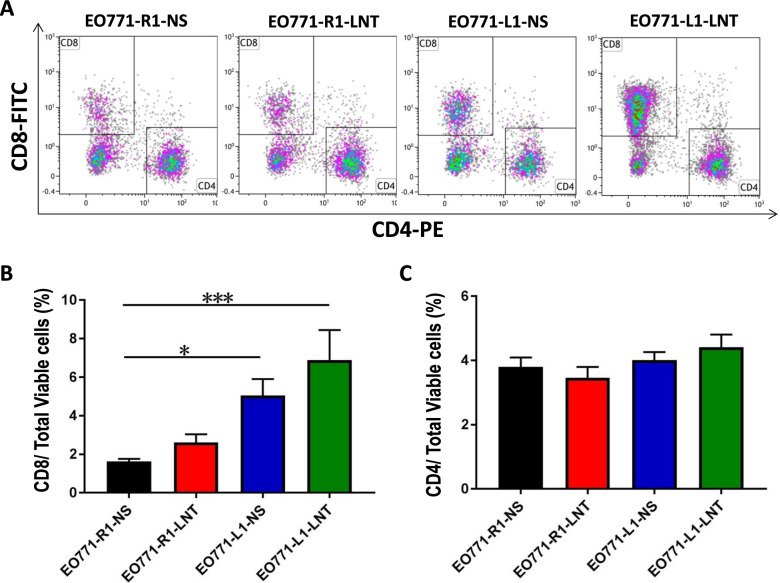
The combination of LNT treatments and DLL1 overexpression increased intratumoral CD8^+^ T cells in EO771 breast tumors. **a** The representative flow figures of CD4^+^ and CD8^+^ T cells in EO771 breast tumors; **b**, **c** The proportions of tumor-infiltrating CD4^+^ and CD8^+^ T cells. All data in this report are presented as means ± SEM. * *p* < 0.05, *** *p* < 0.001

Elevated levels of DLL1 in the TME have been shown to suppress EO771 breast tumor growth via CD8^+^ T cell activation [8]. To test whether LNT combined with DLL1 overexpression can enhance the inhibitory effect on the growth of EO771 breast tumor, we orthotopically inoculated EO771 tumor cells with DLL1 overexpression (EO771-L1) or its control EO771-R1, then treated tumor-bearing mice with 1.0 mg/kg LNT every other day for 12 days (Fig. 1a). Consistent with previous report, EO771-L1 grew slower than EO771-R1 (Fig. 1b-c). LNT treatments retarded EO771-R1 tumor growth (Fig. 1b-c). EO771-L1 with LNT treatments grew slower than either EO771- R1 or EO771-L1, showing additive tumor growth inhibition (Fig. 1b-c). We also analyzed the effects of LNT treatments and DLL1 overexpression on tumor-infiltrating immune cells (Fig. S3). Flow cytometric analysis showed that DLL1 overexpression alone increased intratumoral CD8^+^ T cells, and EO771-L1 tumor with LNT treatments had the highest percentage of intratumoral CD8^+^ T cells (Fig. 2). LNT treatments did not alter the proportions of tumor-associated macrophages (TAMs) and Gr1^hi^CD11b^+^ neutrophils in EO771-L1 tumors, compared with control saline treatments (Fig. S4). Together, these data show that LNT enhanced the inhibitory effect of DLL1 on EO771 breast tumor growth.

### LNT treatments synergize with DLL1 overexpression to induce LAP0279 lung tumor regression

**Fig. 3:**
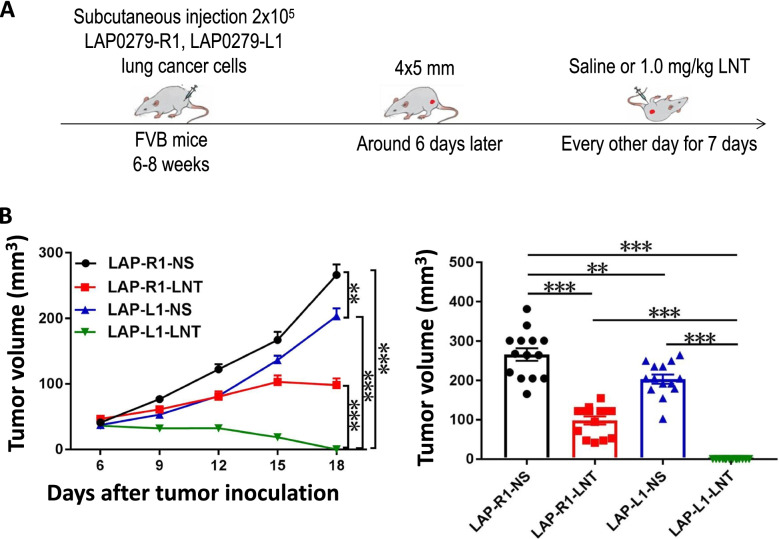
LNT treatments synergistically enhanced tumor growth inhibition of DLL1 in LAP0279 lung cancer. **a** Experimental design: FVB mice were subcutaneously (*s.c.*) inoculated with 2 × 10^5^ LAP-R1 or LAP-L1 lung cancer cells on the right flank. When tumors reached 4 × 5 mm in diameter, mice were randomly divided into 4 groups and received *i.p.* injection of saline or 1.0 mg/kg LNT every other day for 7 days; **b** The growth curves of LAP-R1 or LAP-L1 lung tumors were recorded. Tumor volume was calculated at the end of the treatments. All data in this report are presented as means ± SEM. ** *p* < 0.01, *** *p* < 0.001

The above data show that LNT treatments improve DLL1-mediated tumor growth inhibition in EO771 breast tumor. To determine whether this effect can be seen in other tumor models, we conducted LNT treatments in LAP0279 lung tumor. LAP-R1 and LAP-L1tumor-bearing mice were treated with 1.0 mg/kg LNT every other day for 7 days (Fig. 3a). LAP-L1 lung tumor grew slower than LAP-R1, while LNT treatments significantly inhibited LAP-R1 lung tumor growth (Fig. 3b). Remarkably, three doses of LNT treatments induced LAP-L1 lung tumor regression and tumor kept becoming smaller even LNT treatment was discontinued. Most tumors were disappeared within one week after LNT treatment cessation (Fig. 3b). The data show that LNT treatments synergized with DLL1 overexpression to induce LAP0279 lung tumor regression.

### LNT treatments combined with DLL1 overexpression increase intratumoral CD8^+^ T cells and CD11b^+^Ly6G^+^ neutrophils in LAP0297 lung tumors

**Fig. 4:**
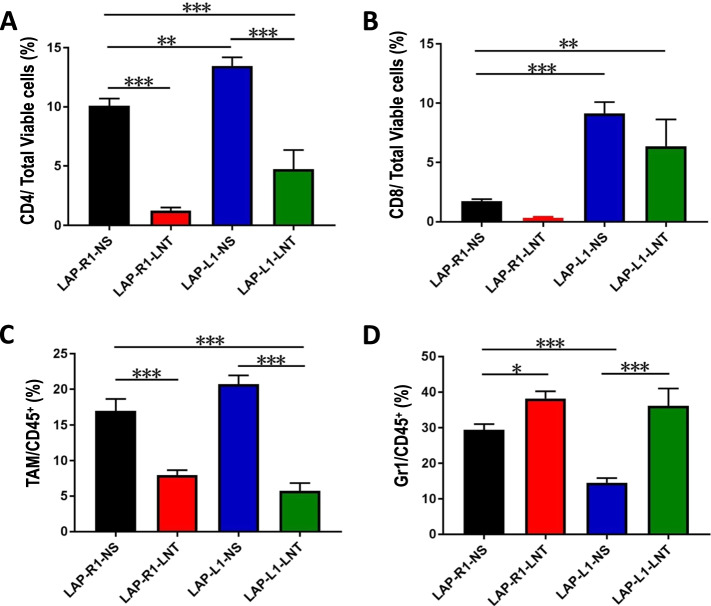
The combination of LNT treatments and DLL1 overexpression in LAP0279 lung tumor simultaneously promoted the accumulation of CD8^+^ T cell and Gr1^hi^CD11b^+^ neutrophils. **a**, **b** The proportions of intratumoral CD4^+^ and CD8^+^ T cells; **c** The proportions of tumor-associated macrophages (TAMs); **d** The proportions of intratumoral Gr1^hi^CD11b^+^ neutrophils. All data in this report are presented as means ± SEM. * *p* < 0.05, ** *p* < 0.01, *** *p* < 0.001

**Fig. 5:**
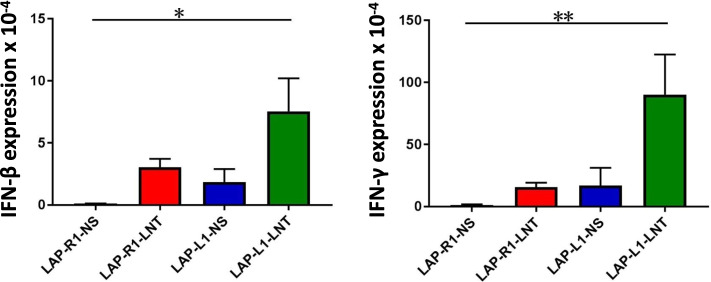
The combination of LNT treatments and DLL1 overexpression in LAP0279 lung tumor upregulated *Ifn-β* and *Ifn-γ* expression. All data in this report are presented as means ± SEM. * *p* < 0.05, ** *p* < 0.01

Since LAP-L1 was very sensitive to LNT treatments, we then started LNT treatments when tumors reached 5–6 mm to obtain tumor tissues for further analysis. We treated tumor-bearing mice with two doses of 1.0 mg/kg LNT and then harvested tumor tissues for flow analysis (Fig. S5 and S6). Flow cytometric analysis showed that LNT treatments reduced the proportions of CD4^+^ T cells and TAMs in both LAP-R1 and LAP-L1 tumors compared with control saline treatments (Fig. 4a and c). LNT treatments increased the proportions of CD8^+^ T cells in LAP-L1 tumors, compared with LAP-R1 tumors (Fig. 4b). Moreover, LNT treatments increased the proportions of CD11b^+^Ly6G^+^ neutrophils in both LAP-R1 and LAP-L1 tumors, compared with control saline treatments, respectively (Fig. 4d). To test whether LNT treatments affect T cell activity, we further analyzed the activation phenotypes of T cells. LNT treatments increased the proportions of activated CD8^+^CD44^+^ T cells in both LAP-R1 and LAP-L1 tumors, compared with control saline treatments, respectively (Fig. S7). LNT treatments also elevated the proportions of CD8^+^CD44^+^CD62L^+^ central memory T cells in both LAP-R1 and LAP-L1 tumors, compared with control saline treatments, respectively (Fig. S8). Consistent with the increase of activated CD8^+^ T cell phenotypes, qPCR analysis data showed that the combination of LNT treatments and DLL1 overexpression upregulated the transcription of *Ifn-β* and *Ifn-γ* in LAP-L1 lung tumor tissues, compared with LAP-R1 tumors with saline treatments (Fig. 5). Together, the data suggest that LNT treatments combined with DLL1 overexpression promote the tumor accumulation of CD8^+^ T cells and CD11b^+^Ly6G^+^ neutrophils, as well as the activation of CD8^+^ T cells in LAP0297 lung tumors.

### LNT treatments enhance the antitumor effects of DLL1 via neutrophils in LAP0297 lung tumors

**Fig. 6:**
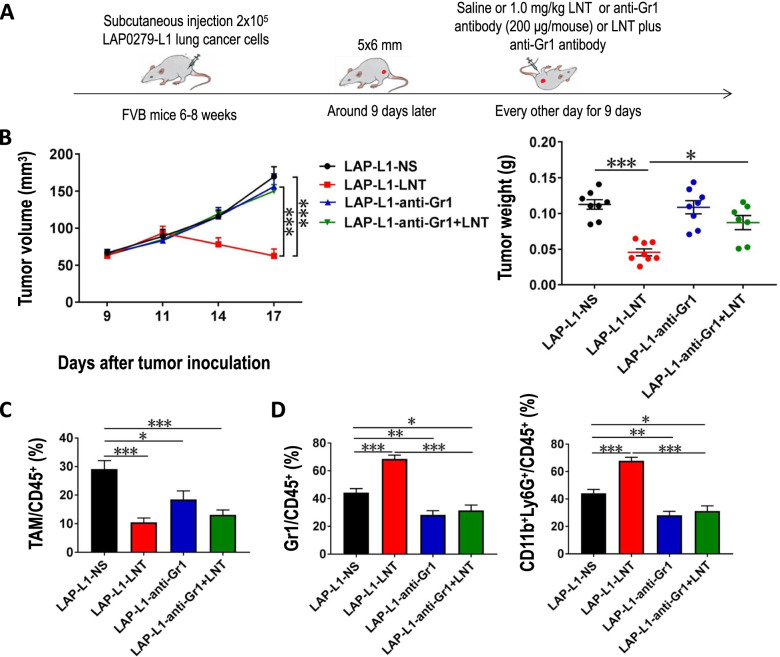
The improved antitumor effects of LNT in LAP-L1 lung cancers were dependent on intratumoral CD11b^+^Gr1^hi^ neutrophils.** a** Experimental design: FVB mice were *s.c.* inoculated with 2 × 10^5^ LAP-L1 lung cancer cells on the right flank. When tumors reached 5 × 6 mm in diameter, mice were randomly divided into 4 groups and received *i.p.* injection of NS (saline), LNT (1.0 mg/kg), anti-Gr1 antibody (200 μg/mouse) or LNT plus anti-Gr1 antibody for 9 days; **b** Tumor growth curves were recorded and tumor weight was measured at the end of the treatments; **c** The proportions of TAMs; **d** The proportions of intratumoral Gr1^hi^CD11b^+^ (CD11b^+^Ly6G^+^) neutrophils. All data in this report are presented as means ± SEM. * *p* < 0.05, ** *p* < 0.01, *** *p* < 0.001

Previous studies showed that IFN-γ mediated the antitumor effects of DLL1 and type I IFNs play a central role in granulopoiesis and promote an anti-tumor phenotype in neutrophils [20, 25]. In addition, β-glucan has been shown to induce an anti-tumor phenotype of neutrophils [16]. Thus, we hypothesized that LNT treatments improve tumor growth inhibition of DLL1 via neutrophils. We then treated LAP-L1 lung tumor-bearing mice with an anti-Gr1 antibody or isotype IgG (200 μg/mouse) every other day for 9 days upon LNT or saline treatments (Fig. 6a). LNT treatments rapidly suppressed tumor growth compared with saline treatments (Fig. 6b). Although anti-Gr1 antibody administration alone did not affect LAP-L1 tumor growth, anti-Gr1 antibody administration reversed the effects of LNT on tumor growth inhibition in LAP-L1 lung tumors (Fig. 6b). Similarly, anti-Gr1 antibody administration alone did not affect LAP-R1 tumor growth, anti-Gr1 antibody administration reversed the effects of LNT on tumor growth inhibition in LAP-R1 lung tumors (Fig. S9). We then analyzed the effects of anti-Gr1 antibody administration on intratumoral myeloid cells (Fig. S10). LNT treatments decreased the proportions of TAMs in LAP-L1 lung tumors compared with saline treatments, which were comparable with anti-Gr1 antibody administration group and anti-Gr1 antibody administration plus LNT treatment group (Fig. 6c). LNT treatments increased the proportions of CD11b^+^ Ly6G^+^ or Gr1^hi^CD11b^+^ neutrophils in LAP-L1 lung tumors compared with saline treatments, while anti-Gr1 antibody administration reversed the increase of neutrophils by LNT treatments in LAP-L1 lung tumors (Fig. 6d). Similarly, LNT treatments increased the proportions of CD11b^+^ Ly6G^+^ neutrophils in LAP-R1 lung tumors compared with saline treatments, while anti-Gr1 antibody administration reversed the increase of neutrophils by LNT treatments in LAP-R1 lung tumors (Fig. S9). Together, the data suggest that LNT treatments enhance the antitumor effects of DLL1 via neutrophils in LAP0297 lung tumors.

## Discussion

Cancer immunotherapy can induce durable antitumor immunity, but only in a small percentage of cancer patients. Combinational therapy is considered as a feasible strategy to improve the efficacy of cancer immunotherapy [10–12]. Elevated DLL1 levels in the TME activated CD8^+^ T cells and slowed down tumor growth [8]. In this study, we found that LNT treatments improved tumor growth inhibition in DLL1-overexpressing EO771 breast tumors. Notably, LNT treatments synergized with DLL1 overexpression induced the regression of most of LAP0297 lung tumors. Our findings suggest that LNT could be as an adjuvant to enhance cancer immunotherapy.

LNT is a polysaccharide from the fruit body of *L. edodes* and has been used previously as a biological response modifier [24, 26]. In particular, LNT has been approved as an adjuvant for the treatment of gastric cancer and brought clinical benefits to cancer patients [25, 27]. Our data showed that LNT combined with DLL1 reduced tumor volume of breast cancer and lung cancer. Moreover, our data showed that LNT treatments increased the percentages of intratumoral CD11b^+^Ly6G^+^ neutrophils in DLL1-overexpressing LAP0297 lung tumors. Anti-Gr1 antibody administration reversed the increased of intratumoral CD11b^+^Ly6G^+^ neutrophils by LNT treatments, and meantime abrogated the improved antitumor effects by LNT treatments. The data suggest that LNT may enhance cancer immunotherapy via modulating neutrophils, while all of increased CD8^+^ T cells, increased neutrophils and reduced TAMs may contribute to tumor growth inhibition induced by the combination of LNT treatments and DLL1 overexpression. This is consistent with previous studies, which show that β-glucan, also a kind of polysaccharide, can trained innate immune cells and elicit immune responses to inhibit tumor growth [17].

Immune checkpoint inhibitors, such as anti-PD1 or anti-CTLA4 antibodies, usually block the inhibitory signalings in T cells, or deplete immunosuppressive cells, such as regulatory T cells, and therefore activate T effectors, such as CD8^+^ T cell, to inhibit tumor progression [11, 17, 28, 29]. This therapeutic modality has shown prolonged survival in multiple tumor types. Polysaccharide, including LNT and β-glucan, did not affect T cells, but can trained innate immune cells, such as neutrophils, to suppressive tumor progression. In the TME, neutrophils are often polymorphic nucleus myeloid-derived suppressive cells (PMN-MDSCs), which can inhibit T cell activity [30]. It is conceivable that trained neutrophils could be polarized from an immune suppressor to an immune stimulator. Thus, polysaccharide could be a group of new agents with the potential to combine with immune checkpoint therapy to achieve better therapeutic outcomes.

Our data showed that it is possible that the optimal treatment dose of LNT could be different for different populations of immune cells. The optimal treatment dose of LNT on a specific population of immune cells with respect to their quantity and function could be also different. Indeed, the 1.0 mg/kg treatment of LNT, but not the 4.0 mg/kg treatment of LNT, increased tumor infiltration of increased the proportion of Gr1^+^CD11b^+^ cells which is the most abundant tumor-infiltrating myeloid cell population in LAP0297 lung carcinoma.

LNT treatments improved the antitumor effects of DLL1 in EO771 breast and LAP0297 lung tumor models. It is striking that LNT treatments induced most of tumor regression in DLL1-overexpressing LAP0297 lung tumor. Furthermore, the effects of LNT on tumor growth were mediated with neutrophils, which is different from DLL1 and commonly used cancer immunotherapy, such as immune checkpoint inhibitors, which activate T cells. Therefore, this study suggests that LNT could be a valuable agent to combined with other kinds of cancer immunotherapy to improve therapeutic efficacy.

## Conclusions

Our findings indicates that LNT and DLL1 may induce synergistical antitumor immunity via simultaneous modulating lymphoid and myeloid cell populations regardless of the type of tumor, providing a potential new strategy to potentiate cancer immunotherapy.

## Supplementary Information

**Additional file 1: ** **Fig. S1.** Relatively lower dose of Lentinan (LNT) treatments inhibited EO771-L1 breast tumor growth. (a) Experimental design: C57BL/6 mice were subcutaneously (s.c.) inoculated with 2×105 EO771-L1 breast cancer cells on the right flank. When tumors reached 3×4 mm in diameter, mice were randomly divided into 5 groups and received *i.p.* injection of different doses of LNT (0.5 mg/kg, 1.0 mg/kg, 2.0 mg/kg, 4.0 mg/kg) daily for 9 days; (b) Tumor growth curves were recorded and tumor volume was calculated at the end of the treatments; NS: control group treated with saline. All data in this report are presented as means ± SEM. * p< 0.05. **Fig. S2.** LNT (1 mg/kg, every other day) treatments inhibited EO771-L1 breast tumor growth. (a) Experimental design: C57BL/6 mice were *s.c.* inoculated with 2×105 EO771-L1 breast cancer cells on the right flank. When tumors reached 3×4 mm in diameter, mice were received different doses of LNT (0. 5 mg/kg, 1.0 mg/kg, 2.0 mg/kg) treatments, every other day, for 9 days; (b) Tumor growth curves were recorded and tumor weight was measured at the end of the treatments. NS: control group treated with saline. All data in this report are presented as means ± SEM. * p< 0.05, ** p< 0.01, *** p< 0.001. **Fig. S3.** The gating strategy to analyze intratumoral CD4+ and CD8+ T cells in EO771 breast tumor model. **Fig. S4.** The effects of LNT treatments on intratumoral macrophages and neutrophils in EO771-R1 and EO771-L1 breast tumors. (a) The representative flow figures of myeloid cells; (b) The proportions of TAMs and neutrophils. All data in this report are presented as means ± SEM. ** p< 0.01. **Fig. S5.** The combination of LNT treatments and DLL1 overexpression synergistically inhibited LAP0279 lung cancer growth. (a) Experimental design: FVB mice were s.c. inoculated with 2×105 LAP-R1 or LAP-L1 lung cancer cells on the right flank. When tumors reached 5×6 mm in diameter, mice were randomly divided into 4 groups and received i.p. injection of saline or 1.0 mg/kg LNT every other day for two doses; (b) Tumor growth curves were recorded and tumor weight was measured at the end of the treatments. NS: control group treated with saline. All data in this report are presented as means ± SEM. ** p< 0.01, *** p< 0.001. **Fig. S6.** The gating strategy to analyze intratumoral CD4+ T cells, CD8+ T cells, neutrophils and macrophages in LAP0297 lung tumor model. **Fig. S7.** LNT treatments increased the proportions of effector CD8+ T cells in LAP lung tumors. (a) The representative flow figures of CD8+CD44+CD69- and CD8+CD44+CD69+ T cells in LAP-L1 lung tumors; (b) The proportions of intratumoral CD8+CD44+CD69- and CD8+CD44+CD69+ T cells in LAP-L1 lung tumors. All data in this report are presented as means ± SEM. * p< 0.05, ** p< 0.01, *** p< 0.001. **Fig. S8.** LNT treatments increased the proportions of central memory CD8+ T cells in LAP lung tumors. (a) The representative flow figures of CD8+CD44+CD62L- and CD8+CD44+CD62L+ T cells in LAP-L1 lung tumors; (b) The proportions of intratumoral CD8+CD44+CD62L- and CD8+CD44+CD62L+ T cells in LAP-L1 lung tumors. All data in this report are presented as means ± SEM. * p< 0.05, *** p< 0.001. **Fig. S9.** The improved antitumor effects of LNT in LAP-R1 lung cancers were dependent on intratumoral CD11b+Gr1hi neutrophils. (a) Experimental design: FVB mice were s.c. inoculated with 2×105 LAP-R1 lung cancer cells on the right flank. When tumors reached 5×6 mm in diameter, mice were randomly divided into 4 groups and received i.p. injection of NS (saline), LNT (1.0 mg/kg), anti-Gr1 antibody (200 μg/mouse) or LNT plus anti-Gr1 antibody for 9 days; (b) Tumor growth curves were recorded and tumor weight was measured at the end of the treatments; (c) The proportions of TAMs; (d) The proportions of intratumoral Gr1hiCD11b+ (CD11b+Ly6G+) neutrophils. All data in this report are presented as means ± SEM. * p< 0.05,** p< 0.01, *** p< 0.001. **Fig. S10.** The gating strategy to analyze intratumoral neutrophils and macrophages in LAP0297 lung tumor model. 

## Data Availability

All data generated during this study are included in the figures and supplementary information.
