# Supplementary figures and images for: Lentinan enhances the antitumor effects of Delta-like 1 via neutrophils
Source: BMC Cancer. 2022 Aug 25;22:918. doi: 10.1186/s12885-022-10011-w (PMC9414423; doi:10.1186/s12885-022-10011-w)

**Fig. S1**

**A**

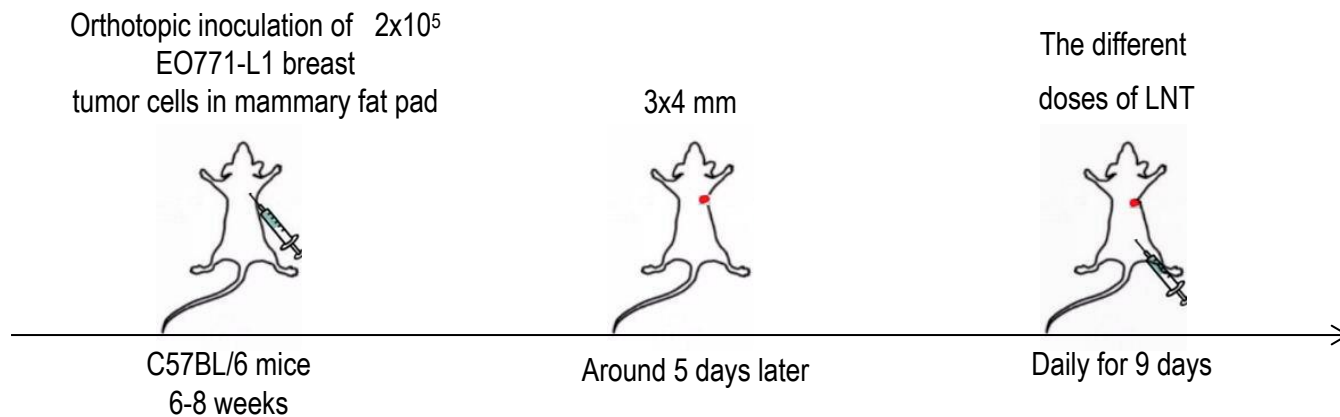

**B**

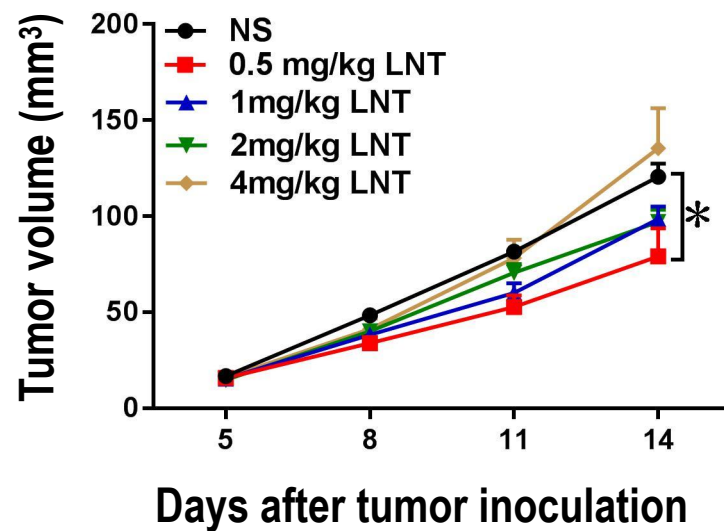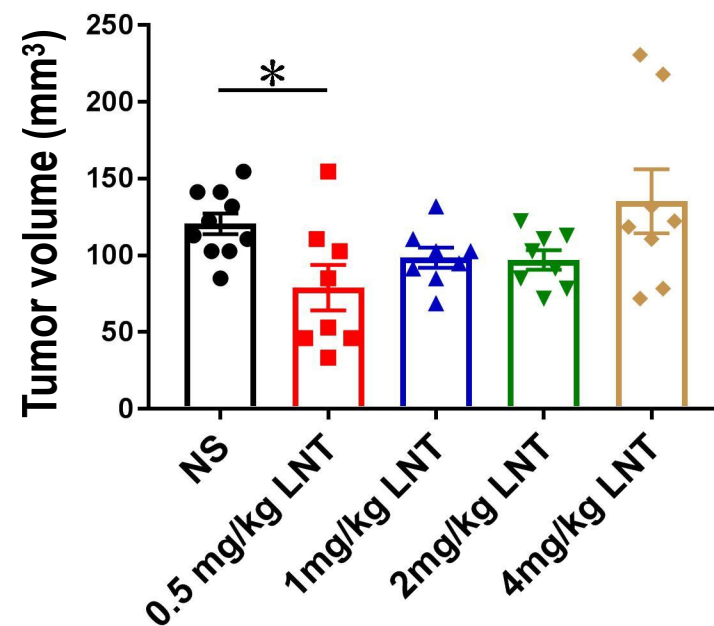

**Fig. S2**

**A**

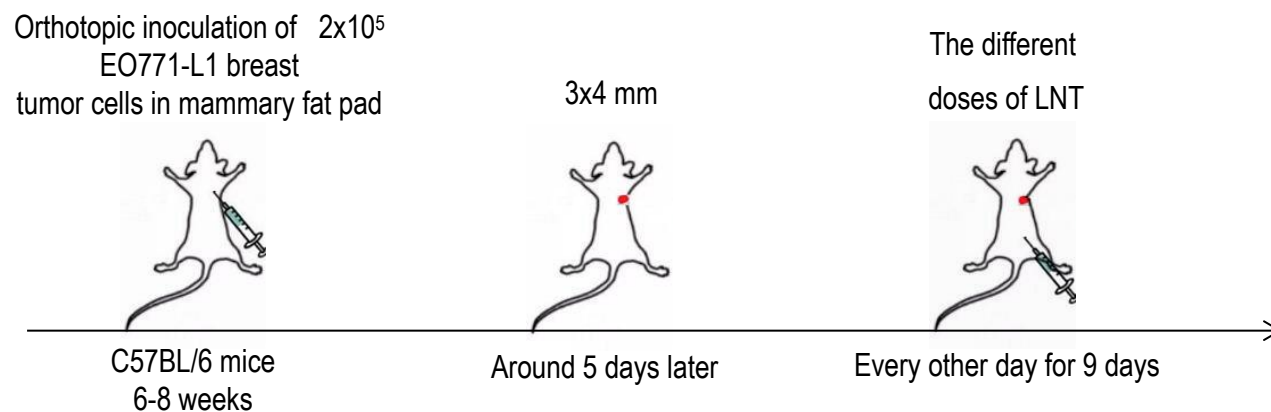

**B**

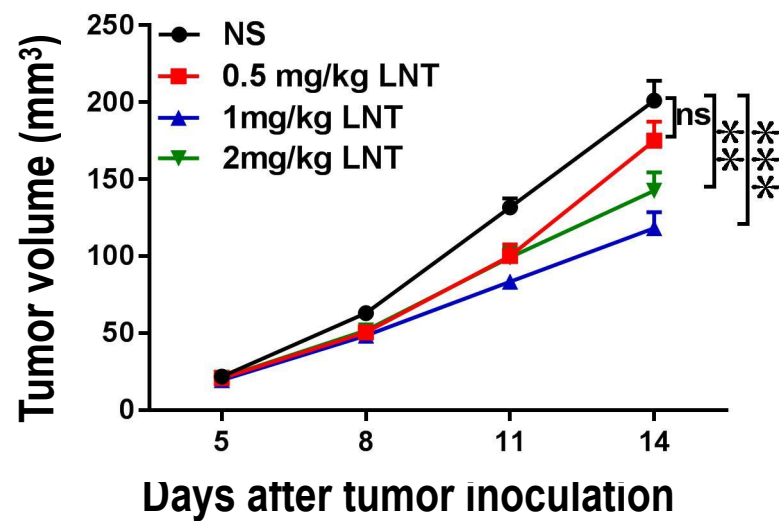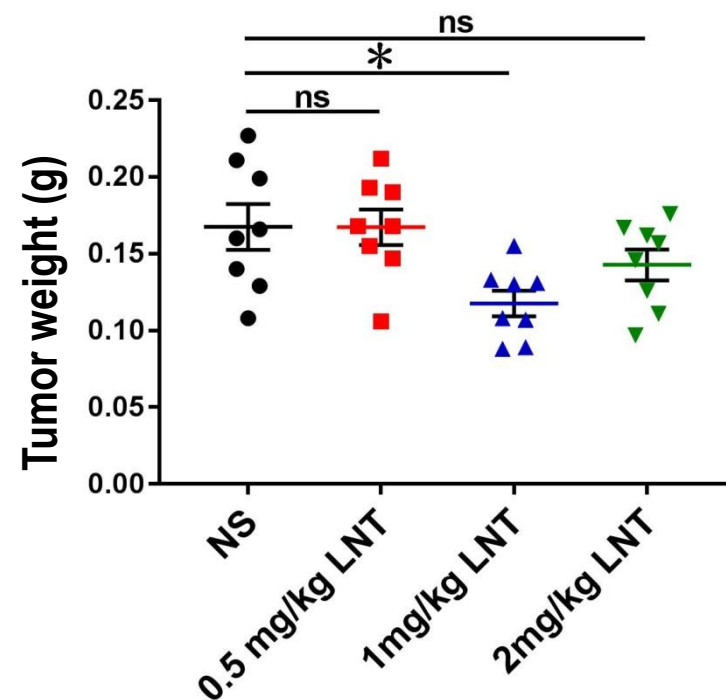

**Fig. S3**

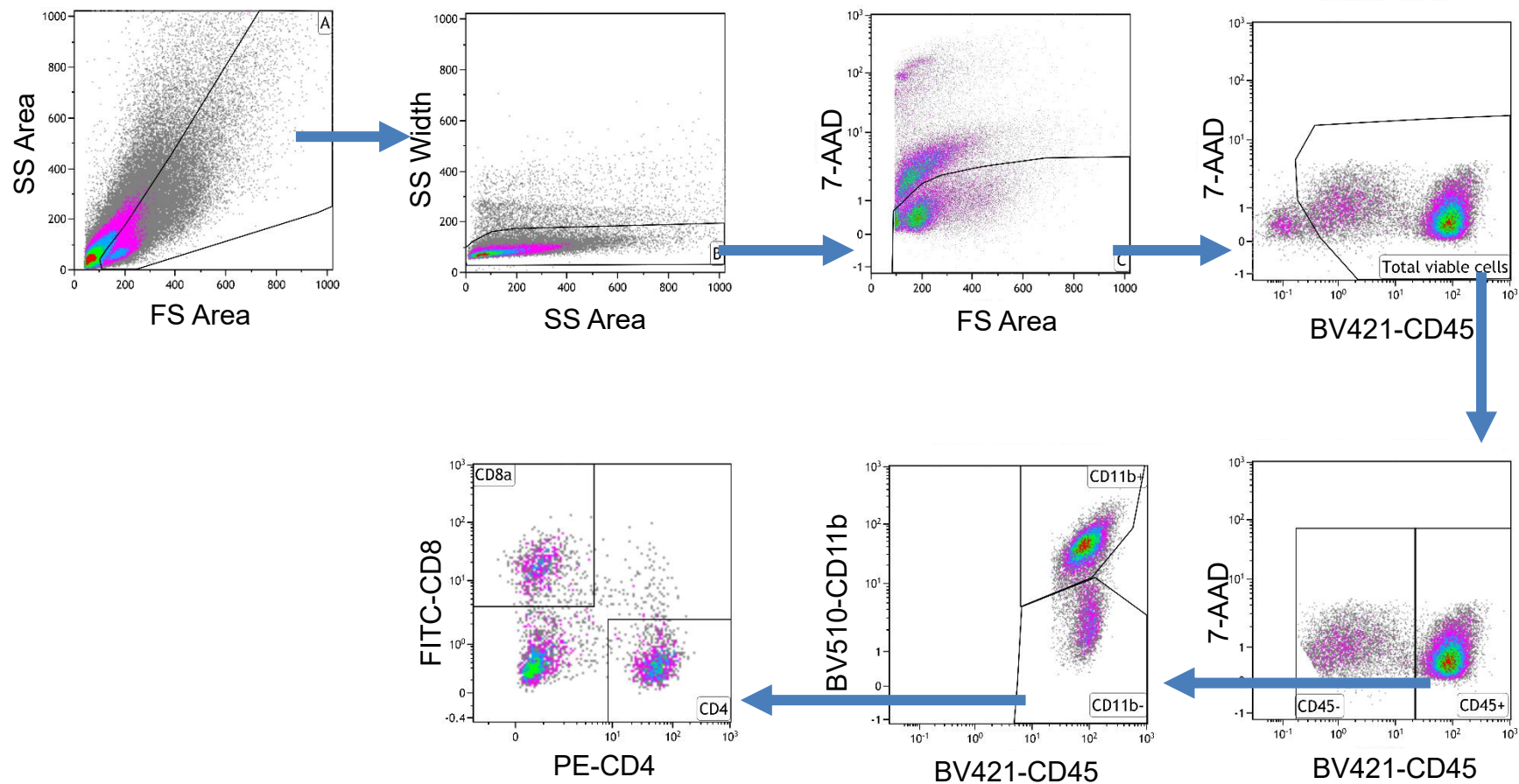

Fig. S4

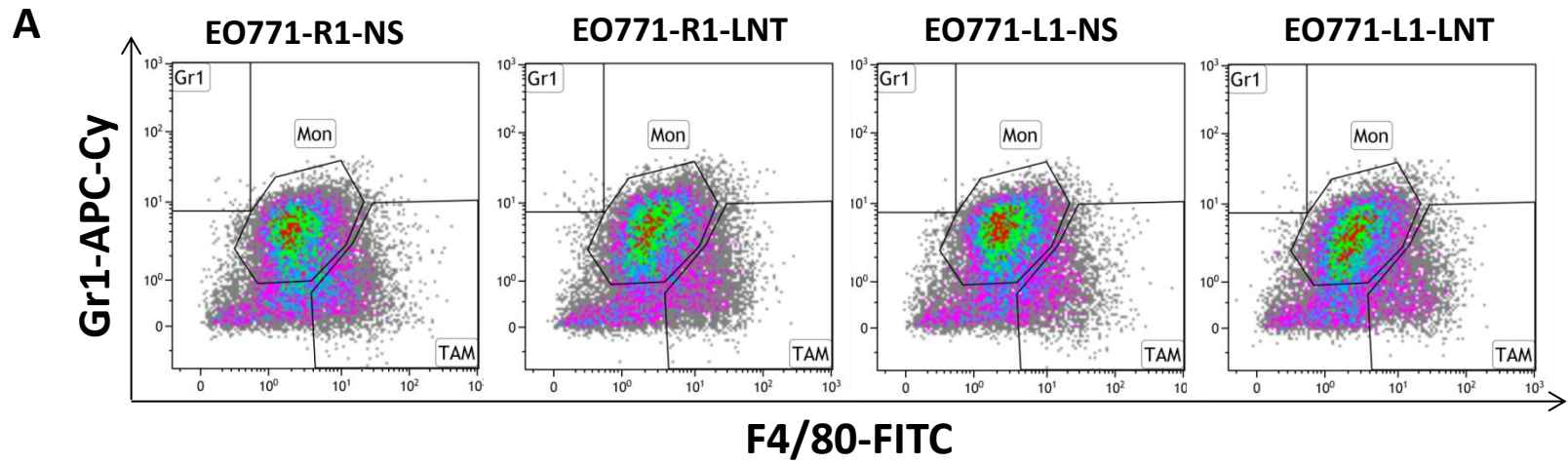

**B**

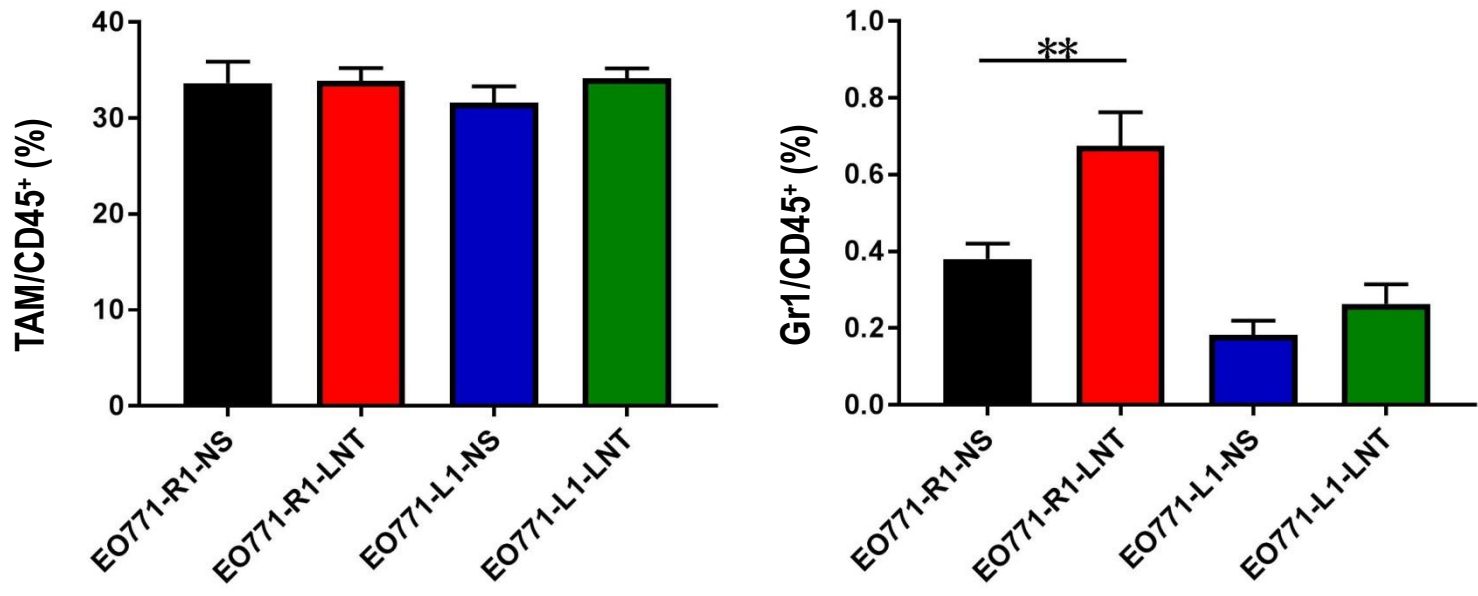

**Fig. S5**

**A**

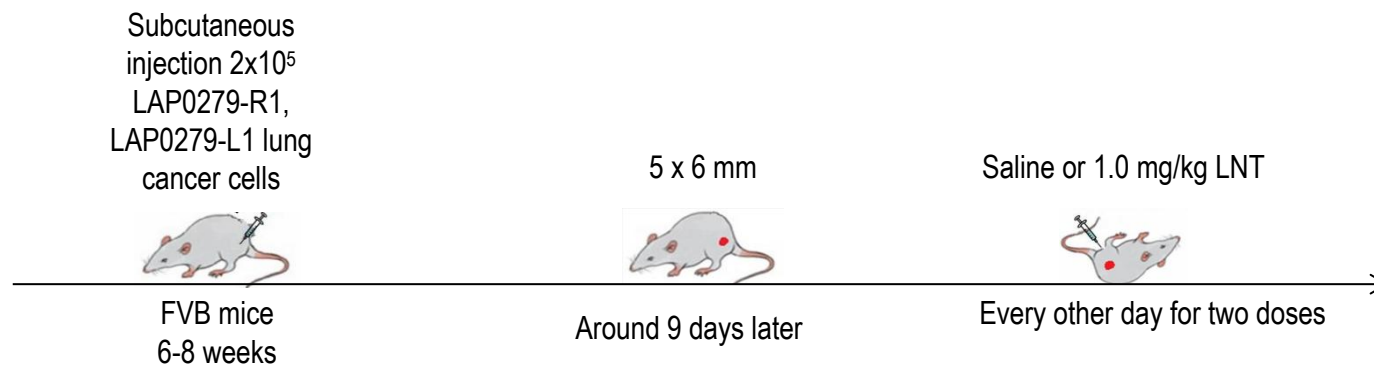

**B**

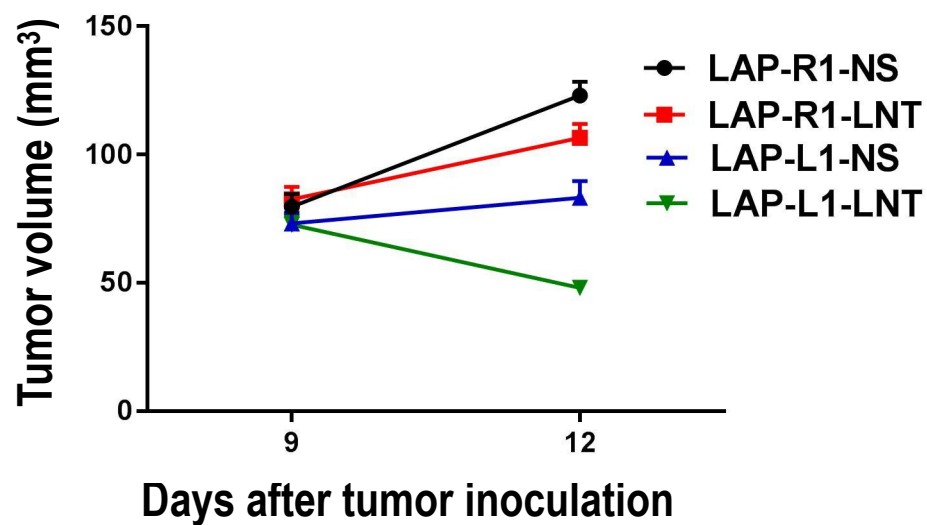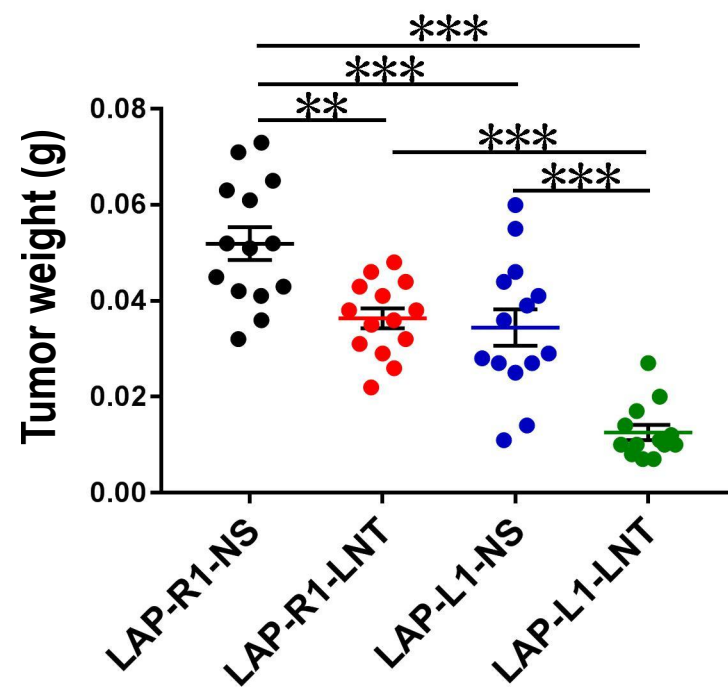

Fig. S6

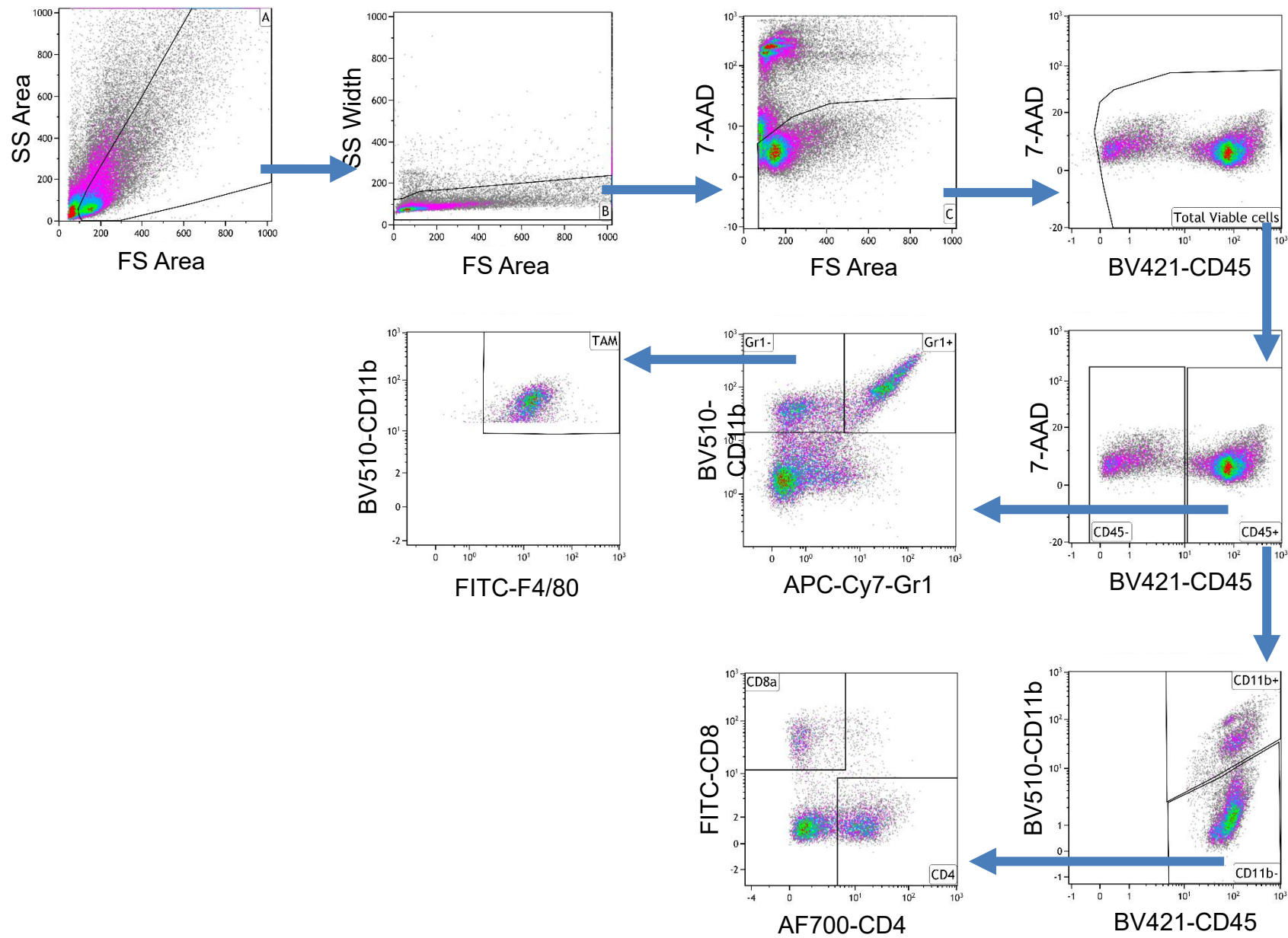

**Fig. S7**

**A**

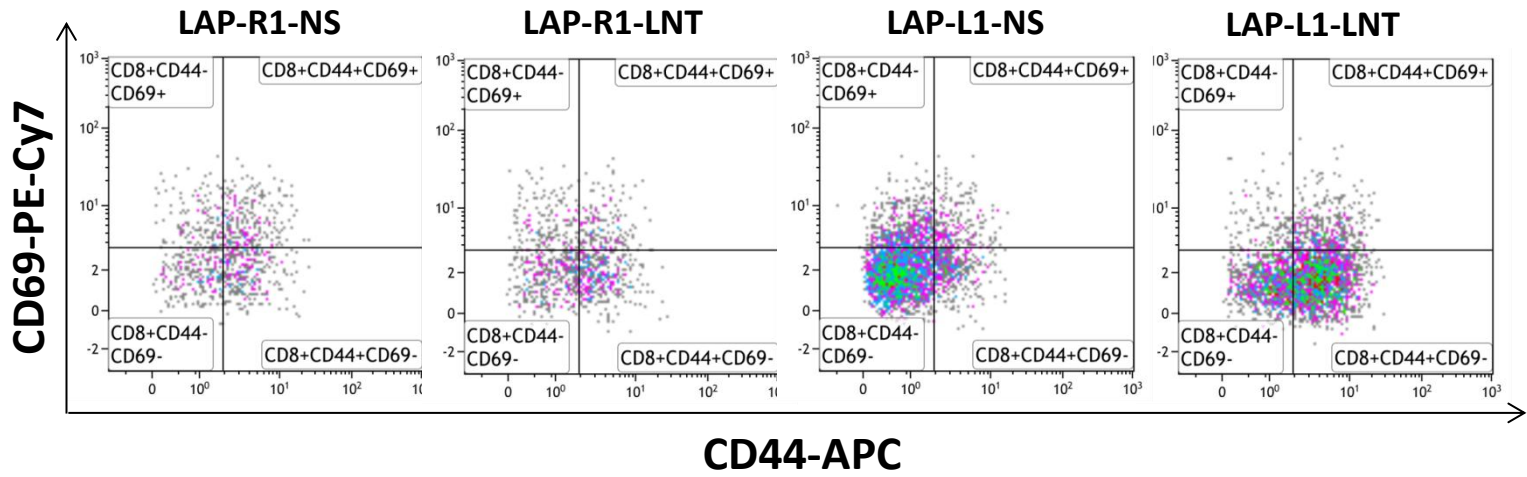

**B**

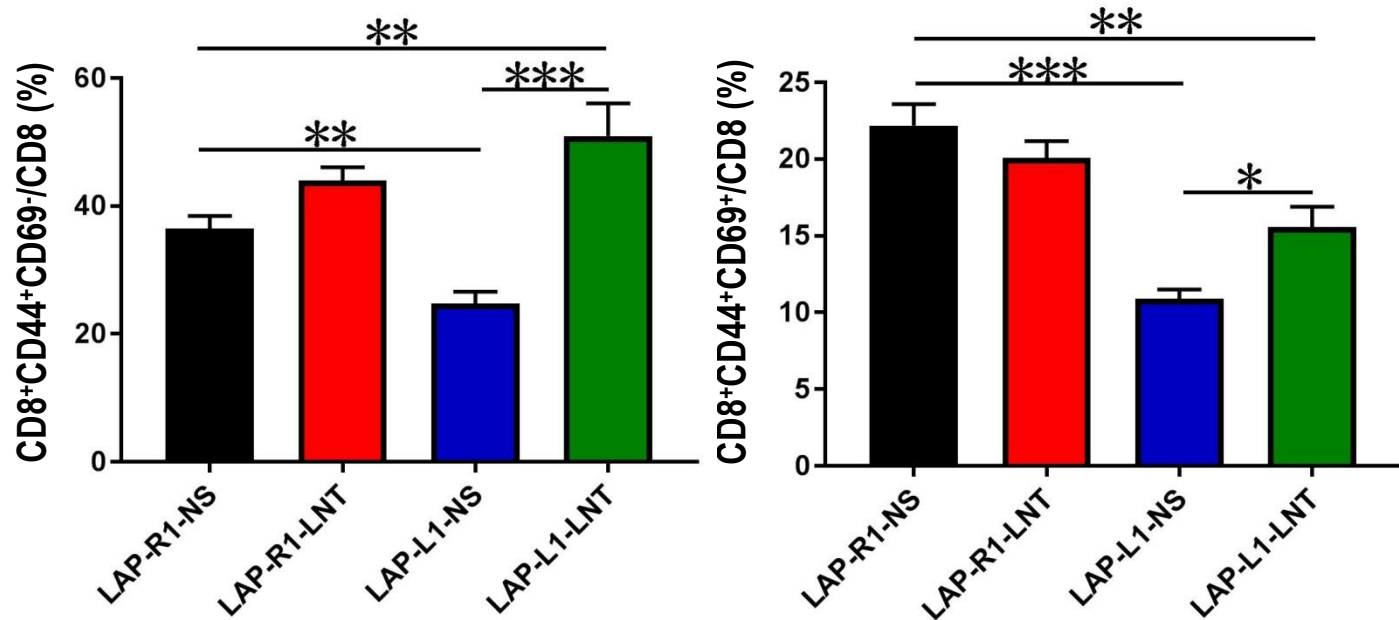

**Fig. S8**

**A**

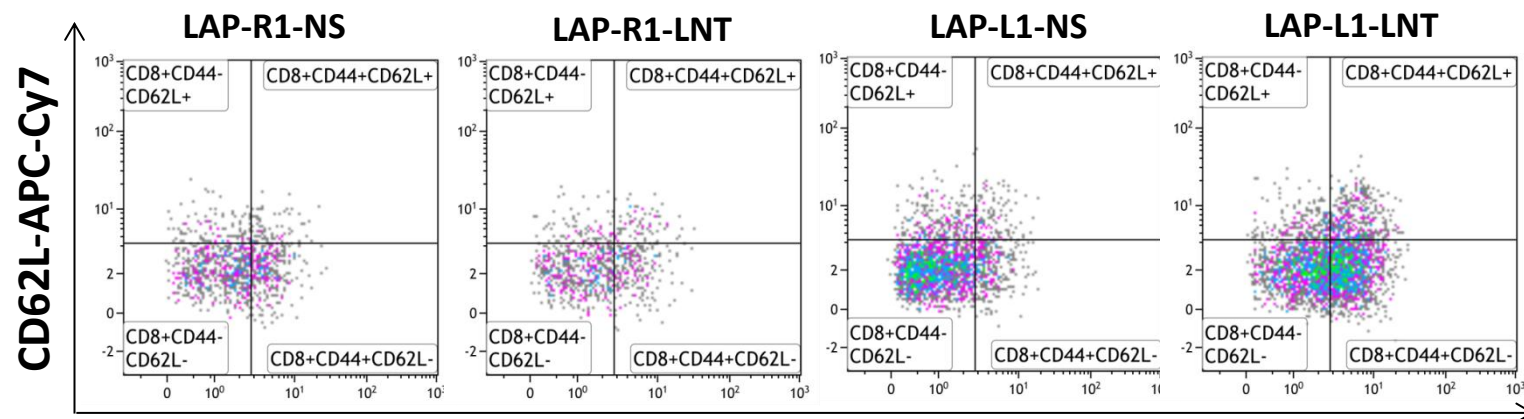

**B**

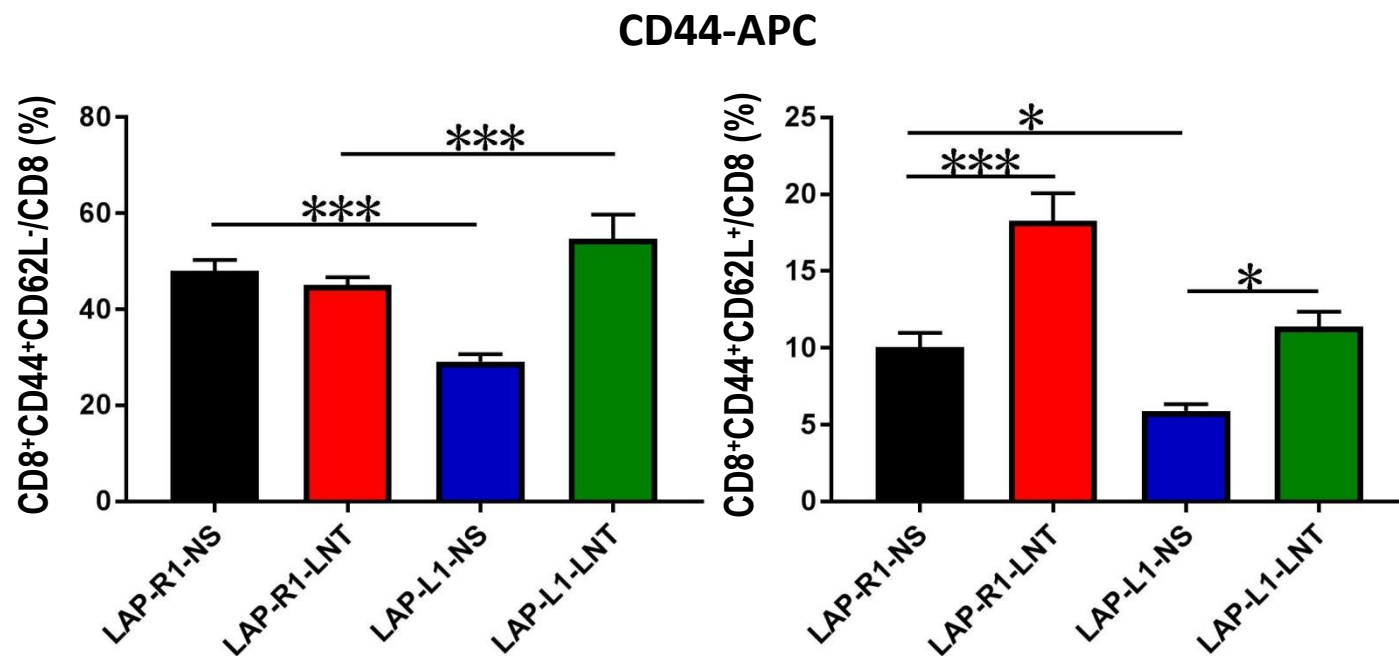

**Fig. S9**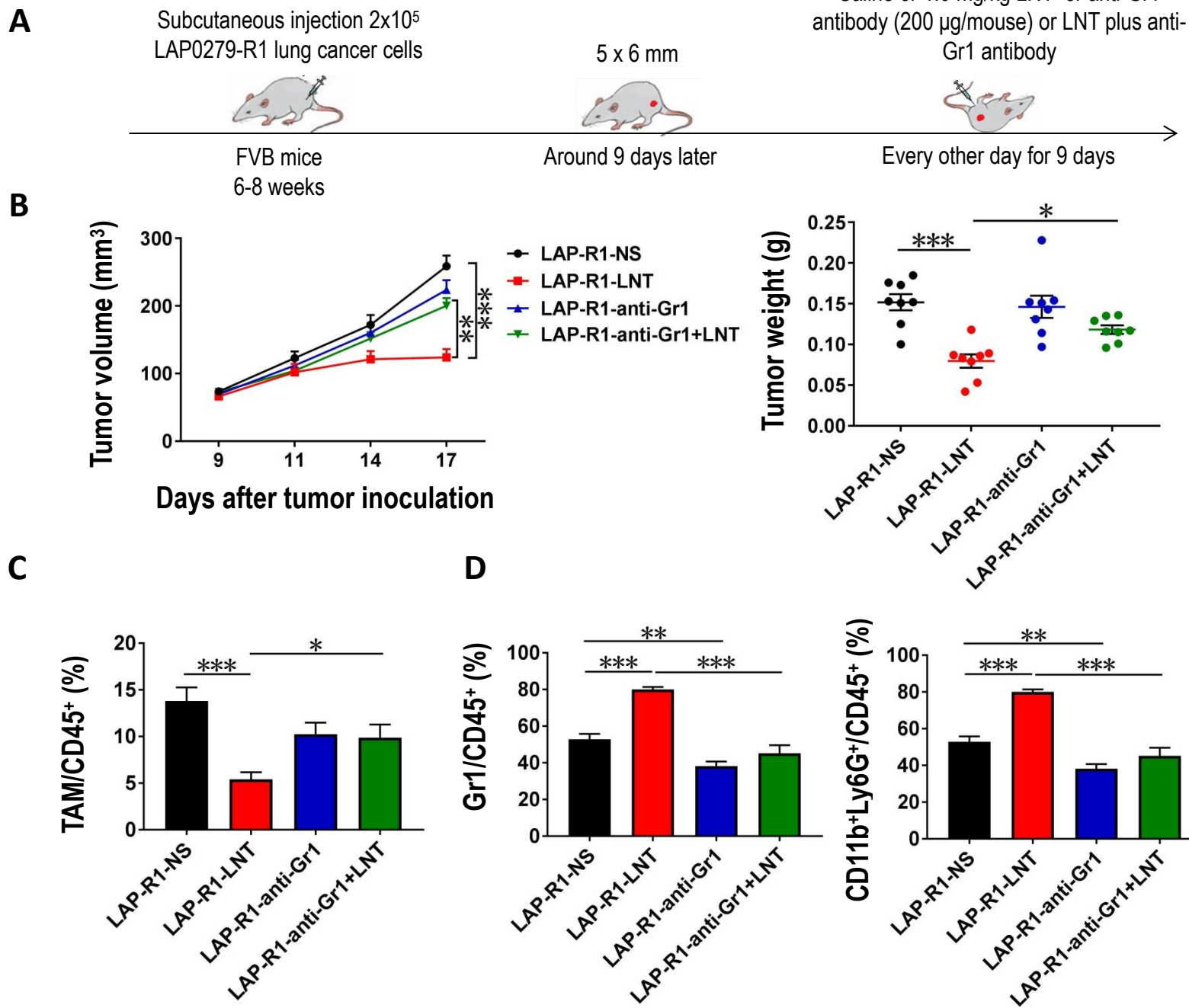

**Fig. S10**

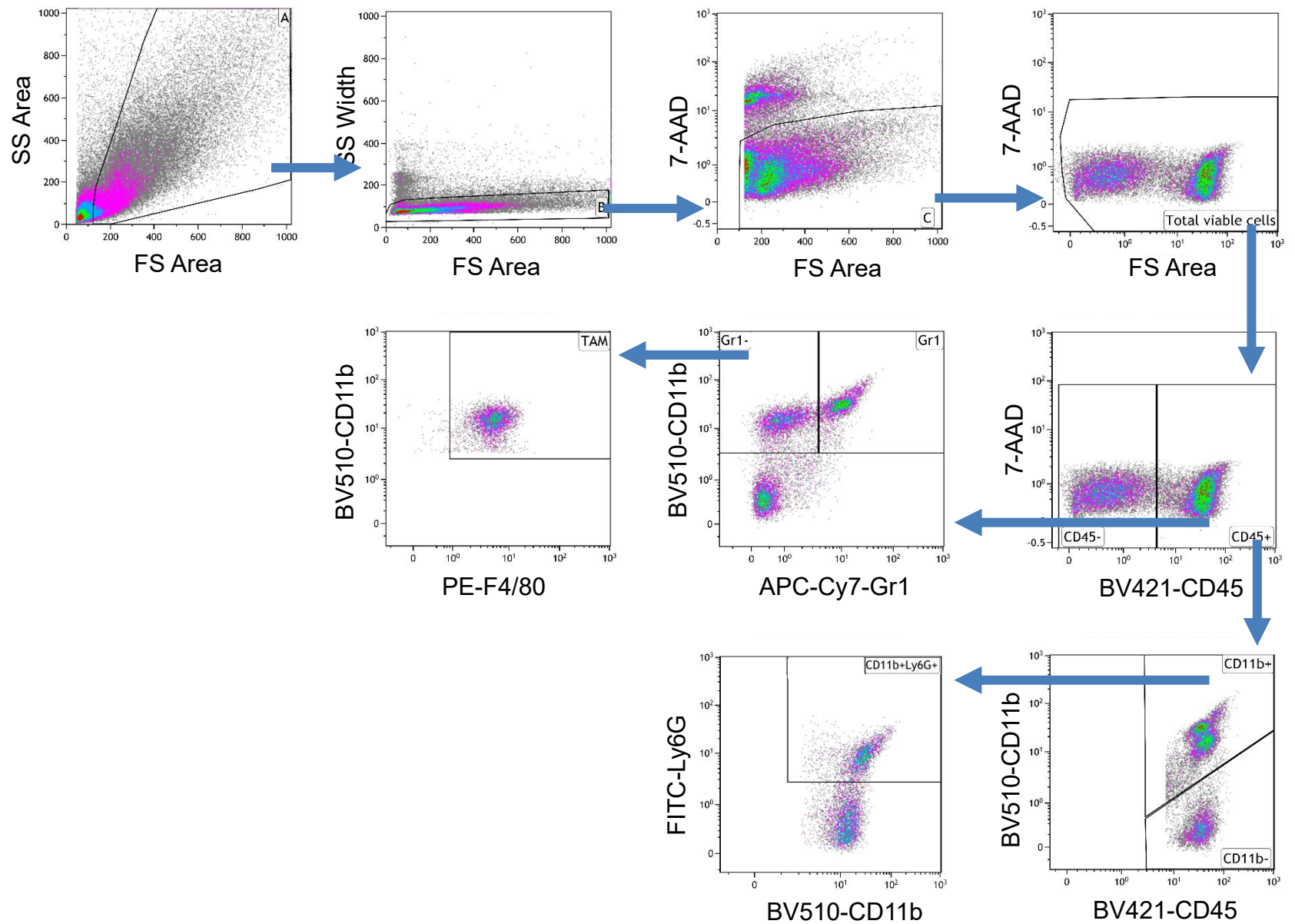

Supplement: Supplementary file 1 — Additional file 1: Fig. S1. Relatively lower dose of Lentinan (LNT) treatments inhibited EO771-L1 breast tumor growth. (a) Experimental design: C57BL/6 mice were subcutaneously (s.c.) inoculated with 2×10^5^ EO771-L1 breast cancer cells on the right flank. When tumors reached 3×4 mm in diameter, mice were randomly divided into 5 groups and received i.p. injection of different doses of LNT (0.5 mg/kg, 1.0 mg/kg, 2.0 mg/kg, 4.0 mg/kg) daily for 9 days; (b) Tumor growth curves were recorded and tumor volume was calculated at the end of the treatments; NS: control group treated with saline. All data in this report are presented as means ± SEM. * p< 0.05. Fig. S2. LNT (1 mg/kg, every other day) treatments inhibited EO771-L1 breast tumor growth. (a) Experimental design: C57BL/6 mice were s.c. inoculated with 2×10^5^ EO771-L1 breast cancer cells on the right flank. When tumors reached 3×4 mm in diameter, mice were received different doses of LNT (0. 5 mg/kg, 1.0 mg/kg, 2.0 mg/kg) treatments, every other day, for 9 days; (b) Tumor growth curves were recorded and tumor weight was measured at the end of the treatments. NS: control group treated with saline. All data in this report are presented as means ± SEM. * p< 0.05, ** p< 0.01, *** p< 0.001. Fig. S3. The gating strategy to analyze intratumoral CD4+ and CD8+ T cells in EO771 breast tumor model. Fig. S4. The effects of LNT treatments on intratumoral macrophages and neutrophils in EO771-R1 and EO771-L1 breast tumors. (a) The representative flow figures of myeloid cells; (b) The proportions of TAMs and neutrophils. All data in this report are presented as means ± SEM. ** p< 0.01. Fig. S5. The combination of LNT treatments and DLL1 overexpression synergistically inhibited LAP0279 lung cancer growth. (a) Experimental design: FVB mice were s.c. inoculated with 2×10^5^ LAP-R1 or LAP-L1 lung cancer cells on the right flank. When tumors reached 5×6 mm in diameter, mice were randomly divided into 4 groups and received i.p. injection of s [file 12885_2022_10011_MOESM1_ESM.pdf]
